# In rats, oral oleoyl-DHEA is rapidly hydrolysed and converted to DHEA-sulphate

**DOI:** 10.1186/1471-2210-7-4

**Published:** 2007-03-09

**Authors:** Marta Serrano, Maria del Mar Grasa, José Antonio Fernández-López, Marià Alemany

**Affiliations:** 1 Department of Nutrition and Food Science, Faculty of Biology, University of Barcelona. Barcelona, Spain

## Abstract

**Background:**

Dehydroepiandrosterone (DHEA) released by adrenal glands may be converted to androgens and estrogens mainly in the gonadal, adipose, mammary, hepatic and nervous tissue. DHEA is also a key neurosteroid and has antiglucocorticoid activity. DHEA has been used for the treatment of a number of diseases, including obesity; its pharmacological effects depend on large oral doses, which effect rapidly wanes in part because of its short half-life in plasma. Since steroid hormone esters circulate for longer periods, we have studied here whether the administration of DHEA oleoyl ester may extend its pharmacologic availability by keeping high circulating levels.

**Results:**

Tritium-labelled oleoyl-DHEA was given to Wistar male and female rats by gastric tube. The kinetics of appearance of the label in plasma was unrelated to sex; the pattern being largely coincident with the levels of DHEA-sulfate only in females, and after 2 h undistinguishable from the results obtained using labelled DHEA gavages; in the short term, practically no lipophilic DHEA label was found in plasma. After 24 h only a small fraction of the label remained in the rat organs, with a different sex-related distribution pattern coincident for oleoyl- and free- DHEA gavages. The rapid conversion of oleoyl-DHEA into circulating DHEA-sulfate was investigated using stomach, liver and intestine homogenates; which hydrolysed oleoyl-DHEA optimally near pH 8. Duodenum and ileum contained the highest esterase activities. Pure hog pancreas cholesterol-esterase broke down oleoyl-DHEA at rates similar to those of oleoyl-cholesterol. The intestinal and liver esterases were differently activated by taurocholate and showed different pH-activity patterns than cholesterol esterase, suggesting that oleoyl-DHEA can be hydrolysed by a number of esterases in the lumen (e.g. cholesterol-esterase), in the intestinal wall and the liver.

**Conclusion:**

The esterase activities found may condition the pharmacological availability (and depot effect) of orally administered steroid hormone fatty acid esters such as oleoyl-DHEA. The oral administration of oleoyl-DHEA in order to extend DHEA plasma availability has not been proved effective, since the ester is rapidly hydrolysed, probably in the intestine itself, and mainly converted to DHEA-sulfate at least in females.

## Background

Dehydroepiandrosterone (DHEA) is quantitatively the main steroid hormone -or prohormone [1]- produced by the adrenal glands in humans, and is secreted mainly as DHEA-sulphate [2]. But in rodents this adrenal production is unclear [3]. In addition to its role as androgen (and estrogen) precursor [4], DHEA is a key neurosteroid [5], antiglucocorticoid [6], metabolic regulator and controller of hormone action [7-9].

DHEA is widely consumed as a drug for a wide range of expected therapeutic actions, including androgen synthesis [10], improvement of degenerative diseases [11] and the treatment of overweight and obesity [12,13]. The rapid absorption and disposal of oral DHEA [14] has generated a number of studies and patents focussed on the maintenance of plasma DHEA levels within a therapeutic range in spite of its rapid metabolism/excretion.

There is a number of naturally occurring fatty acid esters of steroidal hormones, including DHEA [15], androgens [16], estrogens [17], and, especially, oleoyl-estrone [18]. Their functions have not been fully elucidated; androgen and estrogen esters have been postulated as deep storage depot for the corresponding hormones [19,20], and oleoyl-estrone is a ponderostat signal [21]. Hormone esters of varied fatty acid moieties have been proposed as the chimaeric products of sterol acyl-transferases [22]. Acyl-DHEA functions are so far unknown, but a number of tissue esterases have been found to hydrolyse these esters [23,24], including hormone-sensitive lipase [25] and other high K_M _wide-spectrum cell esterases [26].

The oleic acid ester of DHEA was previously synthesized by our group, and tested as a potential slimming drug [27], following the finding of oleoyl-estrone, a powerful hormone eliciting the loss of body fat [18]. Oleoyl-estrone and oleoyl-DHEA are structurally similar, the main difference being the aromatic nature of the A ring in the estrone moiety of oleoyl-estrone. Since orally administered oleoyl-estrone is largely taken up intact [28], and is readily incorporated into the circulating lipoproteins [29], largely bypassing the intestinal and liver esterases, we studied whether the oleic acid ester of DHEA was also absorbed largely intact. This would result in the slow release of DHEA in the bloodstream. As a consequence, the present study deals with the fate of oral oleoyl-DHEA and the analysis of its absorption, breakup and disposal.

## Results

**Figure 1:**
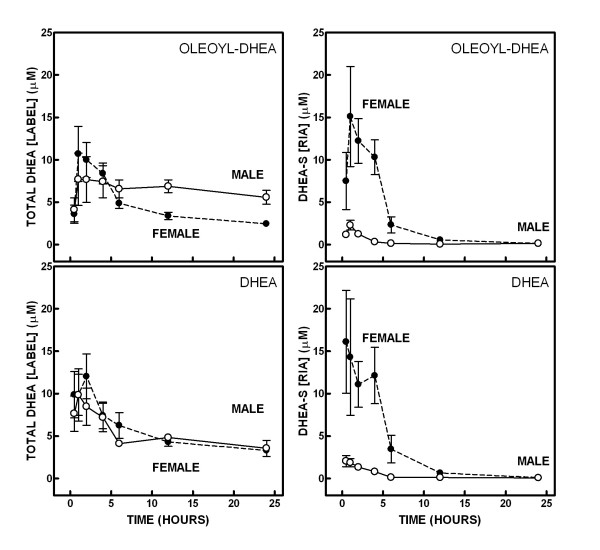
Appearance of DHEA-derived label (left) and levels of DHEA-sulfate (right) in plasma, after the administration of a gavage of tritium-labelled oleoyl-DHEA (upper row) or DHEA (lower row) to male and female Wistar rats. Males: white circles, solid lines, females: black circles, dashed lines. The data are the mean ± sem of 6 different animals. The label data have been converted into equivalent DHEA concentrations using the specific activity of the gavages. The DHEA-sulfate levels were directly measured by radioimmunoassay. Statistical significance of the differences (2-way ANOVAs). The effect of "time" was significant (P < 0.05) for all groups tested. There were no significant differences between sexes for plasma label concentration, and for each sex there were no significant differences either between both types of gavage. There were differences (P < 0.001) between sexes in both oleoyl-DHEA and DHEA gavage-groups for DHEA-sulfate levels, but no significant differences were found for each sex group as to the type of gavage administered.

Figure 1 presents the time-course of appearance of DHEA label and DHEA-sulfate in the plasma of rats given a single oral dose of tritium-labelled oleoyl-DHEA or DHEA. Total DHEA label in plasma was statistically indistinguishable for both sexes and gavages. No differences were found, either, in the patterns (anova) of plasma DHEA-sulfate for both gavages. In female rats, the height of DHEA-sulfate peak was more marked than in males, both under DHEA and oleoyl-DHEA gavages.

However, in both sexes, a difference was observed in the pattern of appearance of DHEA-sulfate between the DHEA and oleoyl-DHEA gavages: in DHEA rats, the 30-min data were higher than those at 1 h, whereas in those receiving oleoyl-DHEA, the values were lower at 30 min than half hour later. This delay may be explained by the timing of oleoyl-DHEA hydrolysis to DHEA and its incorporation into DHEA-sulfate.

In females, the peak of plasma radioactivity, at 1 h after gavage, was coincident with that of DHEA sulfate, both label and the sulfate levels decaying rapidly, with low values from 6 h onwards. The presence of label at 24 h was maintained, albeit very low. In spite of the different systems of calculation, the similarity of the pattern and the closeness of the concentrations involved suggest that most of the label recovered in the first hours in plasma corresponds to DHEA-sulfate at least in females. In males, plasma label DHEA equivalents were higher than the corresponding levels of DHEA-sulfate, irrespective of the gavage being DHEA or oleoyl-DHEA.

**Figure 2:**
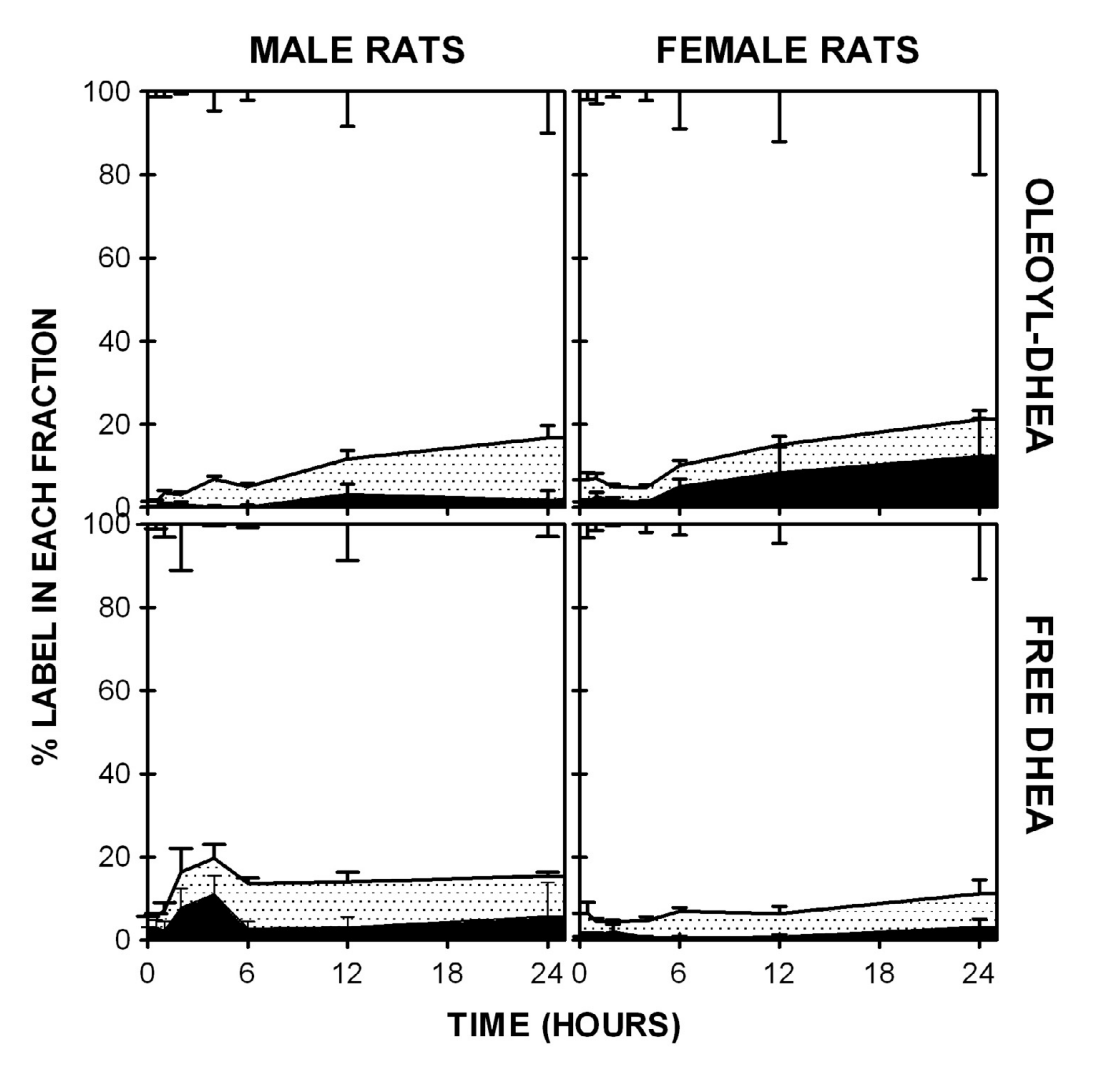
Percentual distribution along time of the oleoyl-DHEA derived tritium label in plasma of Wistar rats (male: left column, female: right column) receiving a single gavage of oleoyl-DHEA (upper row) or DHEA (lower row). Black area: "lipophilic" zone of distribution of the label in a TLC system; dotted area: "intermediate" zone; white area: "hydrophilic" zone. Statistical significance (P < 0.05) of the differences between groups (2-way ANOVAs). For male rats the gavage type resulted in differences for hydrophilic and lipophilic fractions; in females only for the lipophilic. In Oleoyl-DHEA-treated rats, there were no sex-related differences; in DHEA-treated animals sex affected significantly all three fractions.

The distribution of label in plasma (Figure 2) fractions was practically identical for all four groups of animals. In general, a small and variable part of the label (7–15 %) in plasma was found in the intermediate TLC area, and may be attributed to DHEA or to similarly soluble androgen- or estrogen-derived hormones. Most of the label, however could be found in the hydrophilic zone, coherent with a large presence of DHEA-sulfate, but may contain other hydrophilic esters (i.e. glucuronates or sulfates) of estrogens or androgens. Only a small fraction of the label could be found in the form of highly lipophilic compounds (up to 13 % at 24 h in female rats).

Table 1 shows the distribution of oleoyl-DHEA-derived label in selected tissues of the rat 24 h after the gavage. A large part of the label was found in the liver and kidneys, suggesting that their presence was related with direct elimination. The distribution of label (in %; H = hydrophilic; I = intermediate and L = lipophilic) was: liver: males H: 97 ± 1; I: 3 ± 1; L: 0 ± 0, and females H: 97 ± 1; I: 2 ± 1; L: 1 ± 1; kidneys: males H: 95 ± 1; I:4 ± 1; L: 1 ± 1, and females H: 82 ± 2; I: 18 ± 2; L: 0 ± 0; adrenal glands: males H: 96 ± 2; I: 4 ± 2; L: 0 ± 0, and females H: 74 ± 1; I: 15 ± 1; L: 11 ± 3; mesenteric WAT: males H: 88 ± 7; I: 10 ± 4; L: 3 ± 4, and females H: 57 ± 8; I: 25 ± 7; L: 18 ± 7. Skeletal muscle showed much lower label content and a pattern of distribution similar those of liver and kidneys. In all, the tissues analysed accounted for more than 3/4 of the rat weight, but contained, 24 h after the gavage, less than 0.5 % of the initial label.

**Table 1:** Tissue and organ distribution of DHEA label, 24 h after the administration of a single gavage of 35 nmol/g of tritium DHEA or oleoyl-DHEA to Wistar rats

tissue/organ	units	oleoyl-DHEA gavage		DHEA gavage		ANOVA (P)	
		
		male	female	male	female	G	S
liver	nmol/g	3.24 ± 0.93	4.00 ± 1.21	4.30 ± 1.43	5.43 ± 0.87	NS	NS
	nmol	39.3 ± 10.9	31.9 ± 10.0	53.5 ± 18.2	46.1 ± 8.2		
kidney	nmol/g	1.44 ± 0.08*	0.87 ± 0.13	1.33 ± 0.20	1.26 ± 0.12	NS	0.0326
	nmol	1.34 ± 0.16	0.65 ± 0.09	1.27 ± 0.20	0.87 ± 0.06		
spleen	nmol/g	0.40 ± 0.08	0.14 ± 0.02	1.18 ± 0.54	0.26 ± 0.05	NS	0.0439
	pmol	362 ± 48	145 ± 23	846 ± 439	238 ± 53		
adrenal glands	nmol/g	2.35 ± 0.27*	0.59 ± 0.11	1.85 ± 0.22	1.46 ± 0.12	NS	0.0000
	pmol	130 ± 20	54 ± 10	122 ± 26	144 ± 29		
subcutaneousWAT ^1^	pmol/g	96 ± 23	398 ± 179	125 ± 53	192 ± 23	NS	NS
mesentericWAT	pmol/g	86 ± 15	303 ± 85	136 ± 30*	401 ± 58	NS	0.0002
	pmol	137 ± 52	405 ± 111	287 ± 64	567 ± 62		
retroperitonealWAT	pmol/g	53 ± 9	166 ± 37	92 ± 26	134 ± 37	NS	0.0163
	pmol	84 ± 15	252 ± 55	146 ± 44	220 ± 62		
perigonadalWAT ^2^	pmol/g	49 ± 12	120 ± 21	39 ± 11	263 ± 158	NS	NS
	pmol	106 ± 24	470 ± 79	86 ± 24	1123 ± 682		
interscapularBAT	pmol/g	82 ± 13	230 ± 48	185 ± 17	350 ± 95	NS	0.092
	pmol	23 ± 3	54 ± 19	49 ± 5	76 ± 18		
skeletalmuscle ^3^	pmol/g	102 ± 13	180 ± 13	93 ± 13	225 ± 107	NS	NS
	nmol	14.1 ± 1.9	14.5 ± 6.2	12.9 ± 1.8	20.4 ± 9.1		
heart	pmol/g	113 ± 28	93 ± 6	166 ± 78	239 ± 87	NS	NS
	pmol	75 ± 18	56 ± 4	117 ± 47	130 ± 48		
brain	pmol/g	154 ± 30	100 ± 25	178 ± 76	386 ± 129	NS	NS
	pmol	138 ± 27	69 ± 28	196 ± 85	354 ± 104		

^1 ^No data were available for whole-body mass of subcutaneous WAT. ^2 ^Epididymal fat in males and periovaric fat in females. ^3 ^Estimated as 44 % of lean body mass.

The data are the mean ± sem of 6 different animals. WAT = white adipose tissue; BAT = brown adipose tissue. Statistical significance of the differences, 2-way ANOVA; G = effect of "gavage", i.e. differences between oleoyl-DHEA and DHEA; S = effect of sex. Paired-group differences between sex-groups (post-hoc Bonferroni test): * = P <0.05.

The distribution of nonesterified DHEA-derived label in the same tissues showed, in general, a higher label content, but the distribution patterns between the tissues were similar to those obtained with oleoyl-DHEA (no statistically significant differences were found). The presence of lipophilic fractions in the tissues analysed followed, again, a pattern very close to that described for oleoyl-DHEA.

The more marked differences in the distribution of label were found when comparing male and female rats. Liver, heart and brain levels were similar for both genders; males showed higher accumulation of oleoyl-DHEA-derived label in the kidneys, spleen and adrenal glands, while females accumulated more label in the adipose tissues. The pattern obtained from DHEA gavages was similar: no gender differences in liver, kidneys, adrenal glands and heart, higher male levels in spleen and increased label presence in adipose tissues in females.

**Figure 3:**
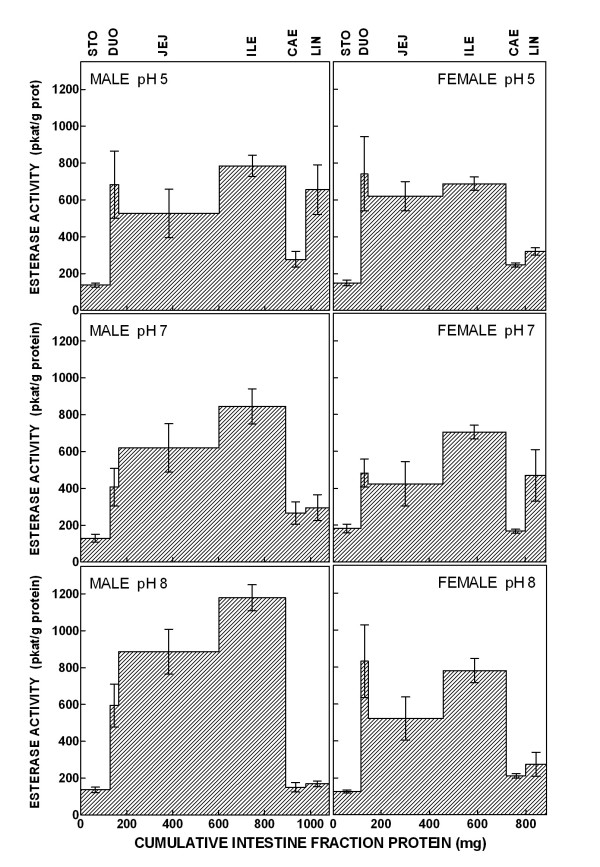
Distribution of the oleoyl-DHEA esterase activity along the alimentary canal in male and female Wistar rats. The measurements were done at three different pH and are expressed in pkat/g of protein; N = 5 for each pH group. The data are plotted against (X axis) the cumulative protein content of the different anatomic sections of the alimentary canal; this way, the surface areas of the space under the curve for each anatomic section roughly correlate with the overall esterase capability of the organ. STO = stomach; DUO = duodenum; JEJ = jenunum; ILE = ileum; CAE = caecum; LIN = large intestine. The statistical comparison of the groups (2-way ANOVAs) showed no significant differences between sexes at different pHs nor differences between different pH for both sexes.

The intestinal oleoyl-DHEA esterase activity at three different pH is shown in Figure 3. Most of the esterase activity was concentrated in the small intestine, with relatively lower levels of activity in the jejunum, and much lower in the stomach and the caecum. The distal part of the intestine shows a esterase capability lower than that of its median part, especially at pH 8. The patterns of esterase activity for male and female rats were fairly similar, both in activity and distribution along the digestive tract. The main differences (not significant) can be found in the relatively lower duodenum activity of males at pH 7 and 8, and the higher large intestine esterase of males at pH 5 and females at pH 7. These patterns are not consistent with the existence of a single esterase activity, and may be more easily justifiable with at least two different enzymes, one working at a more acidic pH and the other with maximal activity at pH 8 or beyond, both presenting different distribution patterns along the alimentary canal.

The liver oleoyl-DHEA esterase activity was similar, but lower, to those of the intestine: 215 ± 77 pkat/g prot at pH 5.0, 362 ± 104 pkat/g prot at pH 7.0, and 596 ± 158 pkat/g prot at pH 8.0 for males and 140 ± 26 pkat/g prot at pH 5.0, 309 ± 48 pkat/g prot at pH 7.0, and 351 ± 71 pkat/g prot at pH 8.0 for females. The large mass of the liver and the obliged transit of the portal blood flow enhance its overall ability to break up the oleoyl-DHEA not hydrolysed by the intestine.

**Figure 4:**
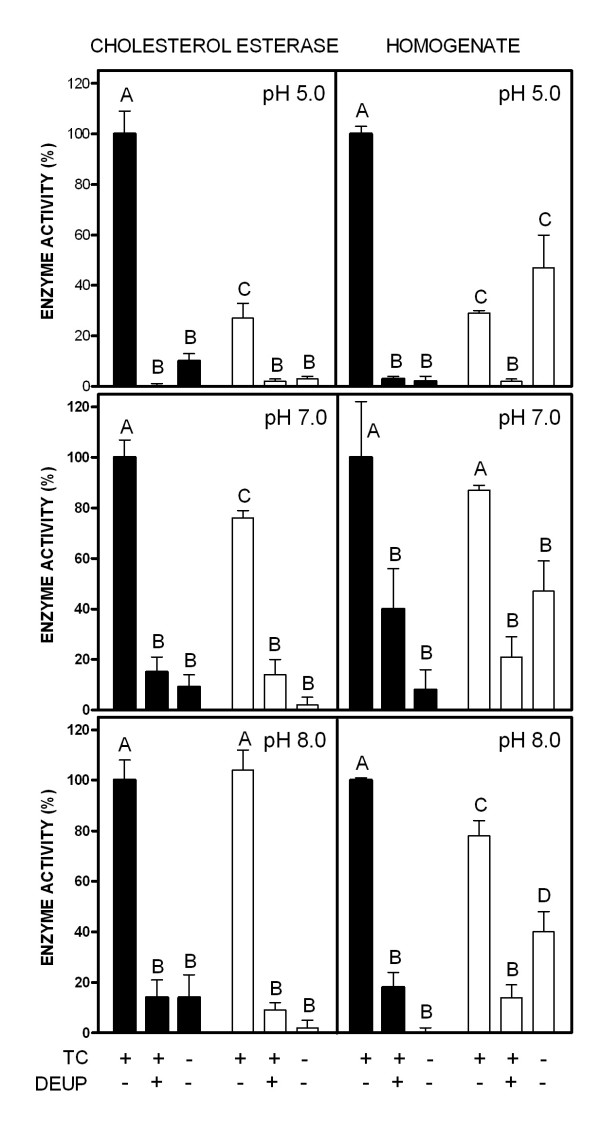
Comparison of the esterase activity of pure hog cholesterol esterase and a female rat duodenum homogenate at three different pH and in the presence of taurocholate (TC) and diethyl-umbeliferyl phosphate (DEUP). The data correspond to the same female rat duodenum homogenates of Figure 3. Black columns present the data obtained using oleoyl-cholesterol as substrate, and white columns those obtained with oleoyl-DHEA. All data are the mean ± sem of 3 different measurements. The columns show the proportionality of enzyme activity under different conditions related to the enzyme activity on oleoyl-cholesterol (100 %). The measured enzyme activities -under the conditions of testing indicated in the text, in the presence of TC and absence of DEUP- for oleoyl-cholesterol hydrolysis were: hog enzyme, pH 5.0: 1.72 ± 0.15 nkat/mg protein; pH 7.0: 3.07 ± 0.22 nkat/mg protein; pH 8.0: 3.04 ± 0.25 nkat/mg protein; intestine homogenate, pH 5.0: 766 ± 23 pkat/g protein; pH 7.0: 454 ± 235 pkat/g protein; pH 8.0: 783 ± 3 pkat/g protein. Statistical comparison between groups (ANOVA): columns bearing different letters are statistically different (P < 0.05).

Pure pancreatic cholesterol esterase acts effectively hydrolysing oleoyl-DHEA. The effects on the DHEA and the cholesterol ester (Figure 4) were slightly different, with higher yields (for the same amounts of enzyme and substrate) in the breakup of cholesterol at pH 5 and 7, but similar at pH 8. In both cases, the presence of taurocholate was essential for the enzyme to work, and the presence of the inhibitor diethyl-umbeliferyl phosphate resulted in a deep inhibition of the esterase for both substrates, an effect more marked at pH 5. The presence of taurocholate was also essential for the breakup of oleoyl-cholesterol by the intestinal homogenate. However, this same homogenate was able to hydrolyse a significant proportion of oleoyl-DHEA in the absence of taurocholate; the proportion of substrate hydrolysed was not different in the absence of taurocholate than in its presence. The inhibition of the homogenate esterase by diethyl-umbeliferyl phosphate was as effective on this preparation as was for the pure enzyme.

## Discussion

The results obtained are consistent with a rapid absorption/hydrolysis of the oral oleoyl-DHEA, since the appearance of label and DHEA-sulfate in plasma is undistinguishable in DHEA or oleoyl-DHEA gavages. However, very shortly after gavage, plasma label was higher in DHEA-treated rats; shortly afterwards, hydrolysis of oleoyl-DHEA rapidly compensated this difference.

Pancreatic cholesterol esterase effectively breaks up oleoyl-DHEA as we have found with a purified hog pancreatic enzyme; its action will take place in the intestinal lumen, past the stomach, and depends on the presence of biliary taurocholate. The remaining oleoyl-DHEA may be broken up by the intestinal wall (tissue) esterase(s) that we have found, acting at a variety of pHs, from the probably lysosomal activity at pH 5 to the lumen and intestinal cell pHs range between 7 and 8. Further hydrolysis of the surviving molecules can proceed when crossing the liver. The consequences are: a very complete and effective hydrolysis of the ester, the immediate absorption of DHEA and its rapid conversion into DHEA-sulfate, which is the main form of DHEA in plasma [30,31]. The excess hormone is probably eliminated via the liver and urine (kidneys) as suggested by the distribution of label, in a way that after 24 h most of the label has been disposed of. This is also in agreement with the known rapid pharmacokinetics of DHEA [32].

Intestinal oleoyl-DHEA esterase activity follows a pattern similar to that described for DHEA sulfotransferase in humans [33]; this association may facilitate the swift transfer of label from the oleoyl-DHEA nucleus into hydrophilic compounds (such as DHEA-sulfate). In rat liver, sulfotransferase activity has been found to be much higher in females than in males [34]; the coincidence of this higher sulfate transfer ability of the female liver with the high plasma peaks of DHEA sulfate, and the lack of gender differences in intestinal sulfotransferase [35], hints at the liver as a main site for the gender-related transformation of the DHEA liberated in the intestine into DHEA sulfate,

The slow relative increase of label in the intermediate phase with time seems to indicate that a small part of the DHEA nucleus ingested has found its way into steroidal hormones -via 3-hydroxy-reductase and aromatase- which are to be found in plasma and in several tissues such as the adrenals. The growing accumulation of label in the lipophilic phase, especially in WAT and adrenals, suggests the conversion and storage of highly lipophilic esters, such as the product of DHEA esterification [36] in adrenal glands. The different level of incorporation of DHEA nucleus-derived label in different WAT sites agrees with their marked differences in steroidal hormone handling [37-39].

The marked differences in label distribution between male and female rats can be traced to the purported different fate of physiological DHEA. In males part of the DHEA is converted to androgen [40], and part continues up to estrone; in females, however, the androgen stage is more limited, with probably most of the DHEA finding its way into estradiol and estrone [39,41]. Since the pharmacokinetics of these hormones in males and females are also different, the final result is a marked sex-related difference in the final distribution of label, both in terms of absolute radioactivity accumulated and the fraction (hydrophilic, lipophilic) bearing the label, consequence of the probably different molecular species into which the DHEA nucleus has derived.

DHEA fatty esters have been found in lipoproteins [22]. In any case, it is improbable that the highly lipophilic fraction of the label progressively appearing in serum will correspond to resynthesized oleoyl-DHEA, since the proportion of label in this fraction increased only when practically no DHEA label was present in serum. This hints at the labelled compound being a lipophilic form (probably a fatty acyl ester) of other steroidal molecular species ultimately derived from DHEA. Lipophilic steroidal hormone esters include androgens [15,16] or oleoyl-estrone [41], which may be recirculated within lipoproteins [29], thus justifying the maintenance of the low persistent presence of label observed in plasma. Since DHEA provides the nuclear structure for the synthesis of both androgen and estrogen in most tissues [39,40,42], and the steroid hormone acyl-ester found so far in highest concentrations in serum is oleoyl-estrone [41], we can speculate that probably the rise in lipophilic label in serum with time may be mainly consequence of label transfer from the DHEA load to the estrone ester.

In spite of the structural similitude with oleoyl-estrone, oleoyl-DHEA was not significantly absorbed as such, and its presence in plasma during the peak absorption period was nil; this means that oleoyl-DHEA was not incorporated into lipoproteins for safe passage from the splanchnic bed to the systemic circulation as occurs with oleoyl-estrone [28].

The results presented suggest that hydrolysis of steroid acyl esters may take place in the intestinal lumen and be effected by a broad-spectrum acyl-cholesterol esterase [43], but also that the intestinal wall contains other esterase(s) different from cholesterol esterase (i.e. may work in the absence of taurocholate, show different pH-related activity patterns), but coincide in their common inhibition by diethyl-umbeliferyl phosphate. The neutral esterase activity may also be related to hormone-sensitive lipase [25]; the recent finding of this enzyme in the intestinal mucosa [44] further strengthens this assumption.

The effectiveness of the combined luminal (i.e. pancreatic), intestinal and liver esterases to hydrolyse oleoyl-DHEA is probably the expression of a powerful and relatively non-specific system of steroid ester hydrolysis that can have a significant influence on the handling, absorption and eventual therapeutic effects of a number of acyl esters of steroidal hormones administered orally.

## Conclusion

The oral administration of oleoyl-DHEA as a form of retarding the plasma availability of DHEA has not been proved effective, since the hydrolysis of the ester proceeds at a fast pace and probably in the intestine itself, making practically undistinguishable the handling of label from DHEA or oleoyl-DHEA after a short initial period.

The overall esterase activity was very similar for male and female rats, in agreement with the rapid hydrolysis of most acyl-DHEA, irrespective of sex.

Incorporation of DHEA into plasma DHEA-sulfate is markedly sex-dependent, hinting at different DHEA handling pathways in males and females. This is supported by the different distribution of label in adipose and other tissues, also influenced by site anatomic and physiological peculiarities.

## Methods

### Animals and oleoyl-DHEA gavage

Wistar rats (Harlan, Sant Feliu de Codines, Spain), 290–350 g males and 200–230 g females, were used. They were maintained in a temperature, humidity and 12 h light cycle controlled environment. The rats had free and continuous access to water and pellet food. All animal procedures were authorized by the Animal Procedures Ethics Committee of the University of Barcelona, and complied with the Catalan, European and Spanish specific rules and constrictions.

Oleoyl-DHEA was custom synthesized (ref. IND6-01-064) by Tecnoquiral (Barcelona, Spain). We synthesized tritium-labelled oleoyl-DHEA from the labelled steroid [1,2,6,7 ^3^H (N); specific activity 2.7 TBq/mmol] (NET-814, Perkin-Elmer, Boston MA USA), and oleoyl-chloride (Sigma, St Louis, MO USA) as previously described for oleoyl-estrone [17]. The product was purified by TLC on silicagel plates (Polygram SIL-N-HR, Macherey-Nagel, Düren, Germany) using hexane/ethyl-ether/acetic acid (20:5:1 v/v). In this system, oleoyl-DHEA Rf was 0.50, DHEA Rf was 0.13 and hydrophilic esters of DHEA (i.e. DHEA-sulfate) did not move from the initial spot. The resulting preparation of labelled oleoyl-DHEA contained less than 1 % DHEA.

Four groups of 6 rats each (2 groups male and 2 female) were used in the experiment. In all animals, the left carotid arteries were permanently cannulated with P50 polyethylene tubing (Beckton-Dickinson, Parsippany, NJ USA) under isoflurane anaesthesia. Four days after the implantation of the cannula, at the beginning of the lighted cycle, the rats were weighed, and given an oral gavage of 0.2 ml sunflower oil containing either 35 μmol/kg DHEA laced with tritium-labelled DHEA (specific activity 3.7 TBq/mmol) [male and female DHEA groups], or 35 μmol/kg oleoyl-DHEA laced with tritium-labelled oleoyl-DHEA (specific activity 100 MBq/mmol) [male and female oleoyl-DHEA groups]. At timed intervals after the gavage, blood was drawn (0.4 ml) from the cannula. Immediately 0.4 ml of sterile saline, followed by 0.1 ml of saline containing heparin were injected through the same cannula in order to prevent hypovolemia and clotting of blood in the tube. Blood was extracted at 30 min, 1, 2, 4, 6 and 12 h after gavage and left to clot to obtain serum; other blood aliquots were kept in dry-heparinized Eppendorf tubes and frozen. Twenty-four hours after the gavage, the rats were killed by decapitation with a guillotine. A final sample of blood was added to the series. The carcasses were then dissected, samples of selected tissues were blotted, cleaned of their contents (stomach and intestines) and frozen with liquid nitrogen until processed. The tissues sampled were: white adipose tissue (WAT) from four different sites: mesenteric, periovarian, retroperitoneal and subcutaneous, interscapular brown adipose tissue, heart, brain, hind leg skeletal muscle, kidneys, spleen and adrenal glands.

### Hormone measurements

Serum samples were used for the ^125^I-radioimmunoanalysis of DHEA-sulfate (kit DSL-3500; Diagnostic Systems Laboratories, Webster TX USA); in this radioimmunoanalysis system, cross-reactivity vs. DHEA was 41 %; since the DHEA fraction (TLC) always contained less than 10 % of label in our samples, we assumed that cross-reactivity with DHEA amounted, at most, to c. 4 %.

Aliquots of 0.2 ml of blood were mixed with 1 ml acetone and vortexed, after centrifugation, the clear supernatants were evaporated in a speed-vac system to dryness. The residues were suspended in 0.100 ml ethanol, of which 0.020 ml were used for tritium estimation by scintillation counting; 0.040 ml were used for TLC separation in three fractions: hydrophilic (Rf = 0; containing, i.e. DHEA-sulfate), intermediate (Rf = 0.13; containing, i.e. DHEA) and lipophilic (Rf = 0.50; containing, i.e. oleoyl-DHEA). The radioactivity in the three TLC bands was also measured in a beta-counter using a standard scintillation cocktail.

Frozen tissue samples (c. 1 g) were homogenized in the cold in 5 ml ethanol/acetone (1:1 v/v) using a Polytron. After centrifugation, the clear supernatants were saved and the protein pellet was again homogenized in 5 ml of ice-cold ethanol-acetone. The combined supernatants were evaporated, and the residue was resuspended in 1 ml of ethanol-acetone; aliquots were used for scintillation counting and TLC.

### Estimation of the intestinal esterase activity

Tritium-labelled 3-oleoyl-cholesterol was synthesized by us from labelled cholesterol [1,2 ^3^H (N); specific activity 1.41 TBq/mmol] (TRK-330, The Radiochemical Center, Amersham UK) using the same esterification and purification procedure than for oleoyl-DHEA. Cold oleoyl-cholesterol (Sigma) was used as control. In the TLC separation procedure, cholesterol had a Rf of 0.40 and oleoyl-cholesterol had a Rf of 0.91.

Undisturbed male and female rats (N = 5 for each sex) were killed by decapitation, and immediately dissected, obtaining samples of liver, stomach, duodenum, jejunum, ileum, caecum and large intestine. The tissues were rapidly blotted, cleaned of their contents, washed in cold saline and maintained in an ice bath. Samples of each tissue were weighed and rapidly homogenized (Polytron) in 10 volumes of ice-cold tris-HCl buffer 0.05 M pH 7.0. Protein content in the homogenates was estimated with the Bradford method [45]. The enzyme activity was measured at pH 5.0, pH 7.0 and pH 8.0; the homogenate was diluted to a protein concentration in the 0.2–0.4 mg prot/ml range, using the buffers: a) acetate buffer 0.1 M pH 5.0; b) tris-HCl buffer 0.1 M pH 7.0; or c) tris-HCl buffer 0.1 M pH 8.0.

Aliquots of 0.100 ml of the diluted homogenates were mixed with 0.125 ml of the same buffer used for dilution, and 0.025 ml of an ethanol solution of either labelled oleoyl-cholesterol (final content: 1.0 nmol/mL, specific activity 9.5 GBq/mmol) or oleoyl-DHEA (final content: 1.0 nmol/mL, specific activity 9.5 GBq/mmol); both compounds were prepared by diluting in buffer a concentrated alcoholic stock solution, the ethanol concentration in the enzyme assay mixtures was, in all cases, lower than 1 g/L.

After mixing, the tubes were maintained at 37°C for 1 hour in a water bath. The reaction was stopped by placing the tubes on ice, and adding immediately 5 ml of trichloromethane/methanol (2:1 v/v). After overnight extraction in an orbital mixer, the organic phase was separated and extracted with 1 ml of 3.9 mM MgCl_2 _for 20 min. The clean organic phase was evaporated to dryness under a gentle stream of nitrogen and the residue was resuspended in 0.200 ml of ethanol. This final extract was used for scintillation counting and TLC estimation of the distribution of the label. All enzyme measurements were carried out in duplicate.

The distribution of the label between the lipophilic and intermediate zones allowed the estimation of the proportion of the labelled substrate initially present that was hydrolysed under the conditions tested. The results were corrected by blanks, in which the homogenate was substituted by buffer, that allowed an estimation of the spontaneous (i.e. non-enzymatic) hydrolysis of the esters. The results were expressed as pkat/g of protein

### Studies using pure cholesterol esterase

A third experiment was carried out comparing the esterase activity of a fresh duodenum homogenate of female rats and a pure cholesterol esterase preparation on both cholesterol- and DHEA-oleoyl-esters. We used a porcine pancreas cholesterol-esterase (C9464, from Sigma) with a nominal activity of c. 15 mkat/g protein. Since cholesterol esterase activity depends on the presence of taurocholate, we tested both activities (pure enzyme and intestinal homogenate) at three pH (5.0, 7.0 and 8.0), in the presence/absence of taurocholate (Sigma; final concentration 6 mM), as well as in the presence/absence of a known cholesterol esterase inhibitor: diethyl-umbeliferyl phosphate (Sigma; final concentration 0.1 mM). The incubation was carried out for only 30 min at 37°C using either oleoyl-DHEA (final content 1.0 nmol/ml; specific activity 5.0 GBq/mmol) or oleoyl-cholesterol (final content 1.0 nmol/ml; specific activity 6.25 GBq/mmol), and adapting the dilution of the pure enzyme and homogenate accordingly. The reaction was stopped introducing the tubes in ice, and the distribution of the label in TLC zones was determined.

Blanks were also included in the analyses, which were carried out in triplicate. The results were expressed as pkat/g of protein.

## Authors' contributions

MA and MMG planned and MMG designed the experiment. MS and MMG carried out the experimental work. MS, MMG and JAFL processed, interpreted the results and drawn conclusions. MA wrote the manuscript; JAFL and MMG critically revised the text. All authors have read and approved the manuscript.
